# Freely Available Training Videos for Suicide Prevention: Scoping Review

**DOI:** 10.2196/48404

**Published:** 2023-11-03

**Authors:** Katherine Wislocki, Shari Jager-Hyman, Megan Brady, Michal Weiss, Temma Schaechter, Gabriela Khazanov, Sophia Young, Emily Becker-Haimes

**Affiliations:** 1 Department of Psychological Science University of California, Irvine Irvine, CA United States; 2 Department of Psychiatry University of Pennsylvania Philadelphia, PA United States; 3 Corporal Michael J Crescenz VA Medical Center Philadelphia, PA United States; 4 Hall Mercer Community Mental Health University of Pennsylvania Health System Philadelphia, PA United States

**Keywords:** freely available videos, asynchronous training, suicide prevention, evidence-based practice, dissemination, implementation

## Abstract

**Background:**

Freely available and asynchronous implementation supports can reduce the resource burden of evidence-based practice training to facilitate uptake. Freely available web-based training videos have proliferated, yet there have been no efforts to quantify their breadth, depth, and content for suicide prevention.

**Objective:**

This study presents results from a scoping review of freely available training videos for suicide prevention and describes a methodological framework for reviewing such videos.

**Methods:**

A scoping review of freely available training videos (≥2 minutes) for suicide prevention practices was conducted using 4 large video-sharing platforms: YouTube, Vimeo, Bing Video, and Google Video. Identified suicide prevention training videos (N=506) were reviewed and coded.

**Results:**

Most content was targeted toward gatekeepers or other lay providers (n=370) versus clinical providers (n=136). Videos most commonly provided content related to suicidal thoughts or behaviors (n=420). Many videos (n=274, 54.2%) included content designed for certain communities or organizations. Less than half (n=232, 45.8%) of training videos included formal clinical content pertaining to assessment or intervention for suicide prevention.

**Conclusions:**

Results suggested an abundance of videos providing broad informational content (eg, “signs and symptoms of someone at risk for suicide”) and a limited portion of videos with instructional content aimed at clinical providers delivering formal evidence-based assessments or interventions for suicide prevention. Development of resources to address identified gaps may be needed. Future work may leverage machine learning techniques to expedite the review process.

## Introduction

Suicide continues to be a pervasive public health crisis, with over 700,000 individuals dying by suicide annually worldwide [1]. Globally, research has indicated that the lifetime prevalence of suicidal ideation is nearly 10%, while the lifetime prevalence of suicide attempts is around 3% [2]. Health care providers play an essential role in delivering evidence-based practices (EBPs) to prevent or reduce suicidal thoughts or behaviors (STB) [3]. Many suicide prevention programs and initiatives also aim to leverage the influence of the general public, lay providers, or gatekeepers (ie, nonmental health professionals who have regular contact with the target population or community; hereafter referred to collectively as “gatekeepers”) in preventing suicide. For example, previous work has demonstrated the role of gatekeepers in identifying those at heightened risk for suicide and connecting them to services [4,5]. Decades of research has emphasized the need for increased training for providers (both gatekeepers and clinical providers) in suicide prevention to maximize the reach of evidence-based suicide prevention techniques to reduce the number of people experiencing suicidal thoughts and behaviors, and ultimately, the number of lives lost to suicide [3,6]. The need to deliver and rapidly scale training opportunities is critical for increasing the number of individuals who can effectively deliver evidence-based suicide prevention techniques [3,7,8].

Effective suicide prevention efforts comprise a mix of strategies, including community education, assessment, and intervention (see [9,10] for reviews of leading evidence-based suicide prevention strategies). Several modalities, including workshops, exist to train gatekeepers and clinical providers in these strategies. In-person, workshop-based training is a prominent method for suicide prevention training. While workshop-based training alone is not sufficient for successful implementation [11], it is often considered a necessary step toward increasing the uptake and use of EBPs. Importantly, previous work has illustrated the importance of leveraging multiple modalities to deliver EBP training to support behavior change, such as the use of video-based skill demonstrations and in-person experiential education or one-on-one hybrid consultation following in-person workshops [11,12]. Unfortunately, the cost and time associated with workshop-based training can be prohibitive for organizations and providers. Training in a new EBP can cost providers (ie, clinicians or organizations) thousands of dollars, and costs escalate when including the consultation and support required for behavior change [13]. These costs are often infeasible, particularly for those in the public mental health system with limited funds [14,15]. An additional barrier to accessing training in suicide prevention EBPs is the limited number of programs that provide training on this topic [16].

It is imperative that the barriers to traditional training approaches are addressed to increase the accessibility of suicide prevention training. Previous work has identified digital approaches, including e-learning, as promising alternatives to traditional in-person methods of training [11,17,18]. Web-based training holds promise as a way for providers to access evidence-based training as either standalone training or as part of a broader training effort (ie, in support of training workshops and graduate training). Further, research assessing web-based training has indicated that it can be potentially comparable to in-person methods of training [11,18]. For suicide prevention specifically, results from a randomized controlled trial of Collaborative Assessment and Management of Suicidality training formats demonstrated comparable outcomes between asynchronous e-learning (leveraging video content) and traditional, in-person learning [18]. This intervention was only freely available to mental health providers within the US Veterans Administration [18]. However, outside of research on specific digital training interventions, there is a limited understanding of other digital training resources for suicide prevention, particularly those that are freely available to the public.

Freely available web-based training can assist in scaling access to training in suicide prevention EBPs. Free digital training content across health care topics has been associated with several benefits, including user satisfaction [19,20], usefulness [19,21], knowledge [22], and self-efficacy [23]. Notably, however, research assessing freely available web-based content is challenging due to the inherent decentralization of producing, distributing, and hosting web-based training content. Previous work on freely available mental health-related content available on the internet related to assessment and treatment has demonstrated a wide range in the quality of content [24-26]. Further complicating this issue, existing platforms that host or distribute web-based training content largely do not review or examine the content beyond aspects related to terms of services, such as copyright infringement or community safety violations (ie, encouraging harm to self or others) [27,28].

Prior research has focused on web-based training interventions described in academic publications [23,29]. However, this approach is limited, as only a small portion of web-based content is likely to be disseminated or examined through academic journals (ie, such as those developed, disseminated, and assessed in intervention studies that are subsequently published in academic journals). Additionally, the included content in reviews targeting academic sources is a potentially biased sampling of extant web-based training. These reviews may not address or include training videos disseminated by clinicians or nonacademic stakeholders. More work aggregating and reviewing training content directly from sources and end users is necessary to better understand the current landscape.

Identifying the landscape of existing freely available video content for suicide prevention is a critical first step toward understanding how to effectively leverage and disseminate training resources to gatekeepers and clinical providers. In addition, understanding existing resources may indicate gaps that would point to areas for future development. For example, it may be that certain interventions are covered in depth, whereas others are not, highlighting where additional development of freely available training resources is needed. Further, reviewing freely available training videos may provide insight into how to improve freely available training resources. While asynchronous training opportunities make it challenging to include experiential components that increase learning [11], some may include skill demonstrations of interventions in action that may facilitate deeper learning, compared to talking about skills conceptually [12]. Identifying the extent to which freely available training videos include skill demonstrations and other components to enhance learning can inform future development.

We conducted a scoping review of freely available web-based training videos for suicide prevention using 4 large, publicly available platforms: YouTube, Vimeo, Bing Video, and Google Video. Given that this is the first scholarly work on this topic, this scoping review focuses broadly on reviewing training videos related to the prevention and management of suicidal thoughts and behaviors, as well as related concepts like nonsuicidal self-injury (NSSI). Our primary aim was to identify and examine the landscape of freely available web-based training for suicide prevention strategies—across screening, assessment, and intervention. Our secondary aim was to demonstrate a novel methodology for reviewing freely available video training content, which can be leveraged for other content areas. Ultimately, this work will provide critical knowledge to support translation efforts in suicide prevention training by illustrating the landscape of freely available training content and providing support for future research aimed at further understanding the quality of available content and areas where additional development is needed.

## Methods

The 4 web-based sources selected for this search were YouTube, Vimeo, Bing Video, and Google Video. These sources were chosen for several reasons: they collectively host (YouTube, Vimeo, Google Video, and Bing Video) and index (Google Video and Bing Video) billions of freely available videos that are published on the internet, billions of users access these platforms on a daily basis, and there are application programming interfaces (APIs) available for each source that can be used to query and collect data [30-32]. Thus, there is a high likelihood that many freely available web-based training videos would be aggregated through these sources. The initial search took place in March and April 2021.

**Figure 1:**
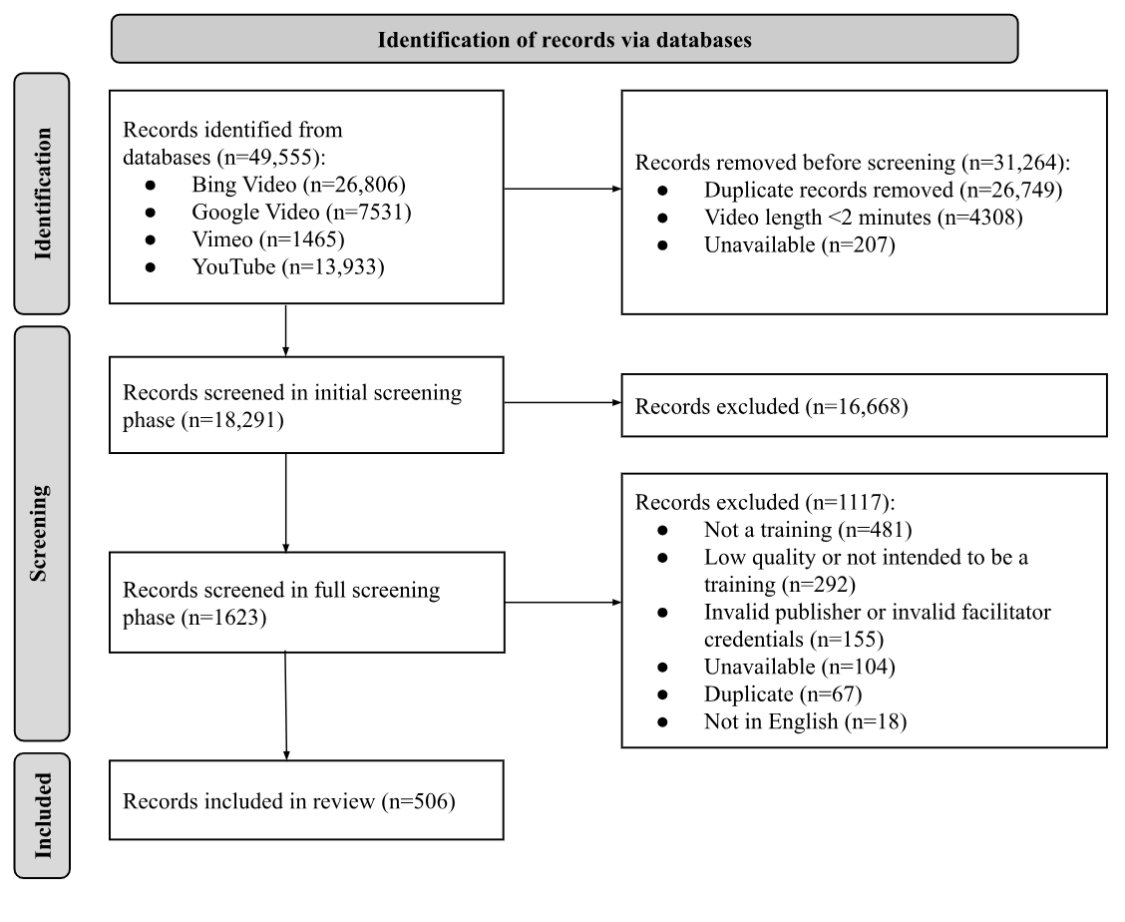
PRISMA (Preferred Reporting Items for Systematic Reviews and Meta-Analyses) flowchart.

Search terms, search strategy, inclusion criteria, exclusion criteria, and content codebook were developed collaboratively by experts in suicide prevention, clinical psychology, and computational social science. Permutations of topic (ie, “suicide” and “self-harm”) and medium (ie, “webinar” and “training”) were made to create each search term. Each search term was queried using Python (Python Software Foundation) and each relevant platform search-specific API [30-32]. Note that a proprietary API search service was used for Google Video. An additional API was used to collect metadata from YouTube results [33] related to our primary inclusion criteria. Guidance for using these resources can be found in the documentation for each specific API [30-33], and the code used for querying each API can be obtained upon request. Our final search terms included: “suicide workshop,” “suicide training,” “suicide education,” “suicide in-service,” “suicide webinar,” “suicide learning,” “suicide online course,” “suicide certification,” “self-harm workshop,” “self-harm training,” “self-harm education,” “self-harm in-service,” “self-harm webinar,” “self-harm learning,” “self-harm online course,” “self-harm certification,” “self-injury workshop,” “self-injury training,” “self-injury education,” “self-injury in-service,” “self-injury webinar,” “self-injury learning,” “self-injury online course,” and “self-injury certification.” Total results (N=49,555) were returned and aggregated across Bing Video (n=26,806), YouTube (n=13,933), Google Video (n=7351), and Vimeo (n=1465). Information including hyperlinks, video identification number, account, length, title, and description was collected. Figure 1 depicts a modified PRISMA (Preferred Reporting Items for Systematic Reviews and Meta-Analyses) flow diagram illustrating the filtering of information through each phase of this review.

The identified videos were required to meet the following criteria to be eligible for inclusion in the final sample: (1) address detection, intervention, or postvention related to suicidal thoughts, suicidal behavior, or NSSI; (2) be free and publicly available; (3) be at least 2 minutes in length (a 2-minute minimum was set to account for existing microtraining sequences; videos less than 2 minutes were deemed unlikely to contain meaningful content); (4) have a working hyperlink; (5) present content in English; (6) have been posted by a government, community organization, academic-affiliated, or otherwise platform-verified source (this was chosen to exclude videos created by lay users such as that for school projects); and (7) have been designed to be delivered through a web-based format (eg, videos that were simply recordings of in-person training were excluded). We also excluded videos that (1) were not predominantly focused on detection, intervention, or postvention related to suicidal thoughts, suicidal behavior, or NSSI (defined as less than 50% of the video content); (2) were interviews with experts; (3) were primarily patient-facing (eg, how to seek help for yourself); (4) included promotional content (eg, advertisements for training with associated costs); (5) did not have distinguishable audio; (6) were news reports or were posted by news organizations; and (7) only included suicide-related content that was limited to describing risk factors without any guidance on how to screen, assess, or intervene with someone at risk.

A total of 3 rounds of initial screening coding were performed by the screening team (EB-H, SJ-H, GK, KW, and MN) prior to the final review and abstraction phase, with overall reliability equal to 80%. Results were deduplicated based on video links, and videos (N=18,291) were split equally between 5 screeners for the primary screening. In the first round of screening (akin to “abstract screening” in a traditional review), the screening team reviewed video titles, brief video descriptions, and author information. Screeners additionally identified clearly ineligible videos.

The second round took place in August 2021, where “maybes” or videos (n=899) in which an inclusion or exclusion decision was not made by a single author were uploaded to Rayyan (Rayyan), a reference management software [34]. In this round, each video was rescreened by at least 2 different authors using Rayyan [34]. Videos in this subset that did not receive either an inclusion or exclusion decision were discussed by authors (EB-H, SJ-H, GK, and KW) through weekly consensus discussions to make final determinations and identify videos that required a full review before eligibility could be determined.

In September 2021, the final phase included a full screening and abstraction of videos (N=1623) performed by MB, TS, and MW. The codebook for abstraction was developed through collaboration between authors. Codes were proposed based on relevance to suicide prevention and feasibility associated with abstracting information for each proposed code. Codes were subsequently refined through weekly team meetings after application to a test subset of videos. Disagreements during the development of the codebook, or other review procedures, were solved through discussion among authors in weekly team meetings. Coding reliability was established prior to screening and abstracting the final sample. Through this process, reviewers achieved good consensus (80% reliability) with a subset of videos (n=66) prior to beginning independent coding. The final sample consisted of 506 videos (1.02% of the initial sample) that met inclusion for review. Coders abstracted relevant information from each video using a structured code sheet (Multimedia Appendix 1). Briefly, we abstracted information related to video content target (STB, NSSI, or a combination of STB and NSSI content), target audience (eg, gatekeeper and clinical provider), content (information about recognizing risk factors and information about treatment, screening, or assessment), whether specialized populations were addressed, video series information (if applicable), and whether the training included any skill demonstrations. Content targets were determined by the focus of the video, such that videos coded as STB include information directed at STB only, videos coded as NSSI offer information directed at NSSI only, and videos coded as a combination of STB and NSSI include information directed at both STB and NSSI. Bimonthly meetings were held among coders, and coders consulted with the senior author (EB-H) to maintain reliability and prevent drift. The full process, from initial screening to abstraction, took place from April 2021 to September 2022. A full list of included videos can be found in Multimedia Appendix 2. This scoping review adhered to the guidelines set by the PRISMA-ScR (Preferred Reporting Items for Systematic Reviews and Meta-Analyses extension for Scoping Reviews) checklist (Multimedia Appendix 3).

## Results

### Overview of Identified Videos

Included videos (N=506) had a mean length of 44.6 (SD 33.03; range 2-175) minutes. As mentioned previously, at least 50% of each included video was dedicated to detection, intervention, or postvention related to suicidal thoughts, suicidal behavior, or NSSI. Most videos were hosted by YouTube (n=365, 72.1%) and intended for gatekeepers or nonprofessional providers (n=370, 73.1%) rather than clinical providers (n=136, 26.9%). Most videos were published after 2018 (n=296, 58.5%), and nearly a third of the videos were published in 2020 (n=155, 30.6%). Training content within the videos was produced by academic institutions or health care facilities (n=179, 31.08%), community organizations (n=211, 36.63%), government entities (n=166, 28.82%), and others (n=20, 3.47%). Of note, videos can be produced by more than one organization.

Roughly half of included videos included content targeted specifically to certain communities or organizations (eg, schools, military, religious communities, and tribal communities; n=274, 54.2%). Similarly, most included videos offered general content, such as warning signs or how to help individuals at risk for suicide more broadly (n=274, 54.2%; hereafter referred to as “broad content”). For example, some of these videos focused on recognizing suicide risk signs and providing information on how to connect at-risk individuals with appropriate care. In contrast, other videos included formal intervention or assessment content (ie, instructional content about clinical assessment and intervention; n=232, 45.8%; hereafter referred to as “formal clinical content”). For example, some of these videos included content focused on delivering formal clinical assessments in response to STB and responding appropriately to STB in various clinical settings. Notably, videos across both categories can be directed at gatekeepers or clinical providers. Several interventions were presented within the latter category, including safety planning (n=63; note this category included interventions referencing an “action plan” or “crisis plan” in addition to the formal Safety Planning Intervention given their similar nature; [35]), postvention (n=29), limiting access to lethal means (n=26), Question, Persuade, Refer (QPR; n=13; [36]), and general coping strategies (n=7). In general, content in these domains tended to consist more of broad overviews of information (eg, the types of information included in a safety plan), rather than in-depth content or illustrations of how to deliver formal clinical content.

Within the videos that included formal clinical content, main content targets included STB (n=204, 87.9%; Table 1), NSSI (n=19, 8.2%; Table 1), or a combination of both STB and NSSI (n=9, 3.9%; Table 1).

**Table 1:** Target audience, video content target, and focus of videos offering formal clinical content^a^.

Target audience and video content target	Total videos, n	Targeted toward organization or system, n	Targeted toward specific lay population, n	Discusses screening or assessment, n	Discusses intervention strategies, n	Skill demonstration, n	Framework for video content, n
Gatekeepers—STB^b^	123	58	55	28	103	8	1
Gatekeepers—NSSI^c^	15	8	9	6	9	0	0
Gatekeepers—combination of STB and NSSI	5	2	2	2	3	0	1
Clinical providers—STB	81	55	N/A^d^	38	58	14	1
Clinical providers—NSSI	4	4	N/A	2	2	1	1
Clinical providers—combination of STB and NSSI	4	3	N/A	4	2	1	0
Total (N)	232	130	66	80	177	24	4

^a^Categories are not mutually exclusive. Videos may receive a code for multiple categories based on video content.

^b^STB: suicidal thoughts or behaviors.

^c^NSSI: nonsuicidal self-injury.

^d^N/A: not available.

### Gatekeeper Videos

Of videos directed at gatekeepers (n=370), most videos offered broad content (n=227, 61.4%) compared to a minority that offered formal clinical content (n=143, 38.6%; Table 1). Some videos were directed at specific populations of gatekeepers, such as educators or school personnel (eg, school staff, teachers, and college or university staff; n=65), caregivers (n=27), military personnel (n=16), students or peers (n=11), and employers (n=11). Videos for gatekeepers provided content mostly focused on STB (n=306, 82.5%), compared to NSSI (n=47, 12.7%) or a combination of STB/NSSI (n=17, 4.6%).

Among the subset of gatekeeper videos that included formal clinical content (n=143), 46.2% (n=66) identified and targeted a specific type or group of gatekeepers, and 47.6% (n=68) were directed at a specific organization or system (Table 1). Of that formal clinical content, a vast majority of videos included intervention content (n=115, 80.4%; Table 1) and only around a quarter of videos included screening or assessment content (n=36, 25.2%; Table 1). Many of these videos varied in the degree to which clinical content was covered, with fewer videos offering in-depth instruction. Notably, only 8 (5.6%) videos included skill demonstrations (Table 1), and only 2 (1.4%) videos included a framework to support the presented content (eg, Cornell University Mental Health Framework, Public Health Action for the Prevention of Suicide; Table 1). A portion of videos designed for gatekeepers had content geared toward providing services for specialized populations (Table 2), including children or youth (n=55, 38.5%), veterans or military (n=14, 9.8%), native or indigenous populations (n=3, 2.1%), LGBTQIA+ (lesbian, gay, bisexual, transgender, queer, intersex, and asexual) individuals (n=1, 0.7%), and other groups (n=15, 10.5%). Other groups included specific racial or ethnic groups; individuals experiencing substance use disorder, bipolar disorder, or autism spectrum disorder; individuals who experienced traumatic events; older adult populations; religious groups; individuals with disabilities; sex workers; and families of military service members.

**Table 2:** Target audience, video content target, and specialized populations for videos offering formal clinical content^a^.

Target audience and video content target	Specialized populations				
	Child or teen, n	LGBTQIA+^b^, n	Native or indigenous individuals, n	Veterans or military, n	Other^c^, n
Gatekeepers—STB^d^	47	1	3	8	12
Gatekeepers—NSSI^e^	5	0	0	6	2
Gatekeepers—combination of STB and NSSI	3	0	0	0	1
Clinical providers—STB	19	0	0	5	16
Clinical providers—NSSI	3	0	0	0	0
Clinical providers—combination of STB and NSSI	4	0	0	0	0
Total, N	81	1	3	19	31

^a^Categories are not mutually exclusive. Videos may receive a code for multiple categories based on video content.

^b^LGBTQIA+: lesbian, gay, bisexual, transgender, queer, intersex, and asexual.

^c^Other specialized populations included (from most to least frequently mentioned): specific racial or ethnic groups; individuals experiencing substance use disorder, bipolar disorder, or autism spectrum disorder; individuals who experienced traumatic events; older adult populations; religious groups; individuals with disabilities; sex workers; and families of military service members.

^d^STB: suicidal thoughts or behaviors.

^e^NSSI: nonsuicidal self-injury.

### Clinical Provider Videos

In contrast to gatekeepers, most videos directed at clinical providers (n=136) offered formal clinical content (n=89, 65.4%), and a minority of videos offered broad content (n=47, 34.6%). Videos for clinical providers mostly targeted STB (n=114, 83.8%) compared to NSSI (n=11, 8.1%) or a combination of STB and NSSI (n=11, 8.1%).

Of videos including formal clinical content (n=89), most were targeted to a specific organization or system, such as academic or hospital settings (eg, universities, medical schools, and K-12 schools), government (eg, state health departments and federal organizations), health care settings (eg, emergency departments and primary care offices), community-based organizations (eg, nonprofits and foundations), or clinical settings (n=62, 70%). Most of these videos focused on intervention strategies (n=62, 70%), whereas fewer focused on screening or assessment (n=44, 49.4%). The depth of formal clinical content varied across videos, with few providing in-depth instruction and skill demonstrations (Table 1). A portion of videos for clinical providers dedicated a majority of content (ie, ≥50% of video content) to specialized populations, including children or youth (n=26, 29.2%), veterans or soldiers (n=5, 5.6%), and other groups (n=16, 18%; Table 2). A small subset of videos (n=16, 18%) contained skill demonstrations, and only a few videos contained a framework for the video content (eg, Comprehensive Approach to Suicide Prevention, Polyvagal Model of NSSI; n=2, 2.2%; Table 1).

## Discussion

This scoping review aimed to examine the landscape of freely available suicide prevention training videos and to demonstrate a novel methodology for reviewing free online video training content. To our knowledge, this is the first effort to empirically quantify the landscape of free video trainings related to mental health. Our methods for identifying existing resources proved to be feasible, albeit time-intensive. We focused on suicide prevention in this first effort given the significant public health burden of suicide and the critical need to advance training efforts related to suicide prevention.

Overall, our findings suggest that freely available suicide prevention videos largely focus on STB (as compared to NSSI), general information about suicide risk rather than specific suicide prevention strategies, and are primarily designed for gatekeeper audiences. A vast majority of videos did not provide a framework underlying the presented information. Most videos were published within the last 6 years, with a disproportionate number published in 2020. This trend likely reflects a global shift to digital and hybrid training opportunities during the COVID-19 pandemic.

Our results point to major gaps in the current landscape of freely available suicide prevention training videos. In particular, there is a relative dearth of formal clinical training content (ie, instructional content about delivering clinical assessments or interventions) for clinical providers working with individuals at risk for suicide. Although some videos provided general overviews of EBPs, few presented in-depth implementation guidance or demonstrations. Few videos presented content relating to the use of formal treatments for STB or NSSI, like dialectical behavior therapy, for example. In addition, few videos, across target audiences, focused on NSSI in isolation or in combination with STB. There was also less content directed at certain specialized populations at higher risk of STB or NSSI (eg, children or youth, veterans or military, native or indigenous populations, LGBTQIA+ individuals, and individuals with disabilities). Although a few videos included skill demonstrations to illustrate how to deliver presented interventions, most content consisted of passive, didactic content inconsistent with recommended practices for training.

While the identified gaps may be a function of the search method (ie, focusing on suicide-focused content instead of specifically searching for interventions that can be applied to STB), this is also likely indicative of notable gaps in freely available resources related to suicide prevention. There may be several reasons for the lack of freely available training videos on certain topics, such as the proprietary nature of certain clinical intervention training content (ie, clinical protocols) and training formats (ie, workshops). There are likely other systemic factors that contribute to these gaps as well, such as the lack of incentives for the development and dissemination of freely available training videos for suicide prevention. Nonetheless, addressing these existing gaps is likely critical for improving and scaling up freely available resources for suicide prevention.

This review also demonstrated the feasibility of leveraging APIs to search, aggregate, and facilitate digital content review to bolster dissemination and implementation research. Partnerships between platforms and researchers can provide greater access to content for research purposes [37]. That said, the number of returned videos in this scoping review exceeded expectations, resulting in greater resources being devoted to screening and coding these videos than originally anticipated. Reviews are typically resource-intensive [38]; however, this review entailed sifting through thousands of videos and then reviewing and abstracting hundreds of hours of video footage. To address this, future work aiming specifically to characterize freely available training resources would benefit from leveraging machine learning techniques to expedite the review process. This can be accomplished at numerous steps in the review process. However, prior work suggests it may be most feasible and accurate within certain phases, such as the screening phase [39]. For example, the use of classification algorithms based on video features (ie, title, description, and publisher information) can potentially accelerate the screening process and is an interesting area for future development. Importantly, this has not been previously assessed with reviews of video content, and it should be validated prior to implementation.

Several limitations of this work should be noted. First, the methodology we used may not capture all freely available web-based training content due to the decentralized nature of freely available web-based resources. While we used some of the most robust platforms for web-based content, freely available resources could be hosted or distributed through different platforms (ie, individual blogs and websites) that were not included in this review. However, given the scope of the reviewed content and the number of duplicate videos identified, we believe that this is a generally representative sample of extant freely available videos for suicide prevention. Second, our coding scheme was constrained to factors that could be reliably assessed across videos, which precluded the inclusion of certain factors of interest, such as sponsorship characteristics or cost of video production, that are not routinely included in training videos. Third, this scoping review was limited to videos with English content. Fourth, other exclusion criteria (ie, video length of at least 2 minutes in length and publisher requirements) may have prohibited the inclusion of content focused on suicide prevention techniques. Further work should explore freely available videos for suicide prevention in different languages, from a range of source types, and of varying lengths. Finally, since this was the first review of its kind and followed established methodological guidelines for scoping reviews [40], no coding was done related to the quality of these training videos.

A critical next step in this line of research will be to conduct an appraisal of the quality of existing videos. As a quality appraisal is not typically included in a scoping review [40], more work is needed to thoroughly evaluate the quality of the content presented within freely available training videos for suicide prevention. While training videos that explicitly encouraged harm or harmful practices were excluded as part of this review during the screening phase, we did not limit our search to evidence-based practices. This is a potential limitation for disseminating this content and an important area for future research. Previous work has used different criteria for assessing the quality of video content [19,25], and this may be relevant for future research.

More research is needed to establish a taxonomy for matching specific resources to end users. Importantly, individuals and organizations should screen training videos to evaluate (1) the quality of the specified resource and (2) how this resource may align with their needs. For example, gatekeepers may benefit from using training videos that correspond to their needs in working with specific populations. Given the lack of skill demonstrations we found in our review, the development of new resources would benefit from prioritizing the provision of detailed instruction on EBPs. It is also important to create evidence-based resources for individuals working with populations that are under-addressed in existing videos. These gaps should ideally be filled by individuals and organizations who are qualified to deliver training related to EBPs for suicide prevention. Video developers should strive to collaborate with researchers and end users to create evidence-based resources that fulfill the needs of consumers.

In conclusion, this is the first review of its kind focused on suicide prevention training, and to our knowledge, the first of its kind in the mental health field. Given calls for increased training for both gatekeepers and professional providers, the findings within this review are critical for advancing existing and future research and training efforts related to suicide prevention. The sample for this scoping review was drawn from large publicly available video-hosting platforms that reach a vast audience, including lay and professional health care providers. Providers often use platforms such as these to search and access information [20]. Increasingly, producers are leveraging these platforms to develop and disseminate training content to health care providers [19,20]. Previous work in other contexts has focused on aggregating and reviewing video content from content developers through academic papers or research studies, rather than searching these platforms directly [23,25,29]. By searching these platforms directly, this review targeted content that is consumer-focused and likely not represented in academic literature. Further, several video-hosting platforms were used to obtain a large sample of representative content for this review. Future research may opt to assess video resources by surveying consumers or producers directly, rather than video-hosting platforms. Results from this scoping review provide information on the landscape of freely available video content for suicide prevention. Future work may leverage and adapt this methodology provided to explore other topics.

## Appendix

Multimedia Appendix 1 Codebook for full review phase.

Multimedia Appendix 2 Included videos.

Multimedia Appendix 3 Preferred Reporting Items for Systematic reviews and Meta-Analyses extension for Scoping Reviews (PRISMA-ScR) Checklist.

## Abbreviations

API: application programming interface
EBP: evidence-based practice
LGBTQIA+: lesbian, gay, bisexual, transgender, queer, intersex, and asexual
NSSI: nonsuicidal self-injury
PRISMA: Preferred Reporting Items for Systematic Reviews and Meta-Analyses
QPR: Question, Persuade, Refer
STB: suicidal thoughts or behaviors
