# Supplementary material for: Freely Available Training Videos for Suicide Prevention: Scoping Review
Source: JMIR Ment Health. 2023 Nov 3;10:e48404. doi: 10.2196/48404 (PMC10656652; doi:10.2196/48404)
Supplement: Multimedia Appendix 1 [file mental_v10i1e48404_app1.docx]

Codebook for full review phase.

| **Full Screen Codebook** |
| --- |
| **Video Information**   - Title - Description - Link - Source - Search Term - Channel Title/Creator Name - Length (in Minutes) |
| **Content Target:**   - SIB vs. NSSI vs. Combination SIB/NSSI vs. Unspecified |
| **Is this video targeted to an organization or system (e.g. workplace culture, schoolwide prevention)? YES or NO**   - If yes, specify type of organization (e.g., school, corporate environment, healthcare), if applicable |
| **Is this video intended for clinical providers specifically? YES or NO** |
| **Is this video clearly intended for a gatekeeper or lay audience (e.g., teachers, parents, police officers, firefighters, peers)? YES or NO** |
| **Is there a specified target lay population (e.g., firefighter, teacher)?**   - If so, note it here |
| **Does this video ONLY cover warning signs of suicide or NSSI and/or how to help broadly? YES or NO** |
| **Does this video discuss formal screening or assessment? YES or NO** |
| **If a specific screening/assessment included:**   - Include name of screening/assessment here |
| **Is the screening/ assessment covered for 2+ minutes? YES or NO** |
| **Does this video discuss intervention strategies for SIB or NSSI? YES or NO** |
| **If a specific intervention is included:**   - Include name of intervention here |
| **Is the intervention covered for 2+ minutes? YES or NO** |
| **Is >50% of this video about a specific population (e.g,. Children, racial/ethnic groups)? YES or NO**   - If yes, specify if: Child/Teen - f yes, specify if: LGBTQ+ - If yes, specify if: Native or Indigenous Individuals - If yes, specify if: Veterans/Soldiers - If yes, specify if: Other (Please Describe) |
| **Trainer**   - Name (if available; otherwise write N/A) - Credentials (if available) - Training Organization |
| **Denote whether the site contains any obvious inappropriate content (e.g., tips for committing suicide, graphic video): YES or NO** |
| **If video is inappropriate, denote brief reason why here, otherwise leave blank** |
| **Is this video part of a series (e.g., part 1 of 4, etc.)?** |
| **Would you recommend this video to a friend? YES or NO** |

Abbreviation: SIB = Suicidal Ideation and/or Behaviors; NSSI = Non-Suicidal Self-Injury; Combination SIB/NSSI = Suicidal Ideation and/or Behaviors/Non-Suicidal Self-Injury; N/A = not available.
